# Clinical presentation and management of multisystem inflammatory syndrome in children associated with covid-19: a retrospective observational descriptive study in a pediatric hospital in Syria

**DOI:** 10.1186/s12879-024-09197-0

**Published:** 2024-03-15

**Authors:** Eman Shhada, Hussein Hamdar, Ali Alakbar Nahle, Diana Mourad, Basheer Khalil, Sawssan Ali

**Affiliations:** 1 Pediatric Intensive Care Department, Faculty of Medicine, Children’s Hospital, Damascus University, Damascus, Syria; 2 Faculty of Medicine, Damascus University, Damascus, Syria; 3 Pediatric Department, Children’s Damascus University Hospital, Damascus University, Damascus, Syria; 4 Rheumatology Pediatric Department, Children’s Damascus University Hospital, Damascus University, Damascus, Syria; 5 Pulmonary Pediatrics Department, Children’s Damascus University Hospital, Damascus University, Damascus, Syria

**Keywords:** Multisystem inflammatory syndrome in children, COVID-19 infection, Pediatric infection, Post-infection Syndrome, Retrospective observational study

## Abstract

**Objective:**

Multisystem Inflammatory Syndrome in Children (MIS-C) associated with COVID-19 is a rare and serious medical condition. This study aims to review the clinical presentation, laboratory parameters, outcomes, and management of MIS-C cases in a pediatric hospital in Syria.

**Methods:**

This retrospective observational study aimed to investigate MIS-C between May 2020 and October 2021. Data collection involved extracting information from medical records, and patients were identified based on the case definition established by the World Health Organization (WHO). Various laboratory investigations, diagnostic evaluations, clinical presentations, and treatments were performed to assess patients. Descriptive statistical analysis was conducted using Microsoft Excel.

**Results:**

A total of 232 COVID-19 cases were reported with COVID-19 Infection. Among these cases, 25 (10.77%) were identified as MIS-C. The median age of the patients was 5.5 years, with the majority being male patients (72%). Patients experienced fever (100%), bilateral conjunctivitis (88%), rash (84%), gastrointestinal symptoms (76%), and cardiac dysfunction (72%). Other notable findings included oral cavity changes (64%), edema (36%), cervical lymphadenopathy (36%), and neurological manifestations (28%). Respiratory symptoms were uncommon (16%). All patients recovered, with no recorded deaths.

**Conclusion:**

The predominant presence of positive SARS-CoV-2 IgG in the majority of patients in this study supports the post-infectious nature of MIS-C. Respiratory symptoms were less prevalent in both pediatric COVID-19 and MIS-C patients. Early supportive care is crucial in management, although additional research is needed to establish definitive guidelines. Larger studies are necessary to overcome the limitations of this study and to enhance our understanding of MIS-C in pediatric COVID-19 patients.

## Introduction

Coronavirus disease 2019 (COVID-19), arising from the severe acute respiratory syndrome Coronavirus, has significant implications for the global population. Approximately 20% of individuals infected encounter severe manifestations, resulting in a mortality rate of 2.3% [1, 2]. Although the majority of children afflicted by COVID-19 exhibit mild symptoms or remain asymptomatic, a subset of children may develop critical illness necessitating hospitalization, intensive care, or respiratory assistance [3].

Multisystem Inflammatory Syndrome in Children (MIS-C), a rare yet severe medical condition associated with COVID-19 [4], is characterized by inflammation in various organs, including the heart, kidneys, lungs, brain, skin, eyes, or gastrointestinal system. The causes of MIS-C remain unknown but it has been linked to SARS-CoV-2 infection [4, 5]. About 40–50% of children with MIS-C meet criteria for complete or incomplete Kawasaki disease (KD). Hypotheses regarding the pathophysiology of MIS-C include: (1) an autoimmune-mediated inflammatory process, (2) a cytokine storm represented by superantigen response, (3) a dysfunction in immune response toward chronic exposure to SARS-CoV-2 viral antigen [6].

The clinical presentation of MIS-C may lead to potential misdiagnoses with toxic shock syndrome (TSS), secondary hemophagocytic lymphohistiocytosis, or macrophage activation syndrome (MAS) [7]. Most studies showed that the risk factors associated with developing MIS-C may vary by gender (males), age, and ethnicity (black and Hispanic races) [7–9]. This study aims to comprehensively review and summarize the clinical presentation, laboratory parameters, outcomes and management of MIS-C cases present in pediatric hospital in Syria.

## Materials and methods

### Study design, settings, and variables

Our research focuses on a retrospective observational descriptive study carried out at the University Children Hospital, including patients diagnosed with MIS-C between May 2020 and October 2021. Researchers underwent training sessions on MIS-C prior to data collection, covering its clinical manifestations and relevant aspects. On June 11, 2023, information was extracted from paper-based medical records and subsequently entered into an Excel spreadsheet. Parents were contacted via telephone to validate and provide any missing details, including clinical presentation, laboratory tests, and socio-demographic characteristics.

This study utilized a methodology that involved the collection of data from patients’ medical records, encompassing socio-demographic characteristics, laboratory parameters, clinical presentation, treatment and management approaches, as well as the outcomes in pediatric patients with MIS-C. The identification of MIS-C patients was based on the case definition established by WHO [10]:

- Adolescents and children between 0 and 19 years who presented with fever for 3 days or more.
- Presentation with two of the following: (1) Bilateral non-purulent conjunctivitis, rash, or muco-cutaneous inflammation manifestations; (2) Hypotension or shock; (3) Signs of pericarditis, myocardial dysfunction, valvulitis, or coronary abnormalities with echography findings or elevated Troponin/NT-proBNP); 3) Evidence of coagulopathy such as Partial thromboplastin time (PTT), Prothrombin time (PT), elevated d-Dimers; (4) Acute gastrointestinal dysfunction (vomiting, diarrhea, or abdominal pain).
- Elevated markers of inflammations such as C-reactive protein (CRP) and Erythrocyte sedimentation rate (ESR).
- Absence of infection by any microbial cause other than SARS-CoV-2. Evidence of infection with (SARS-CoV-2) based on real time polymerase chain reaction (RT-PCR) or exposure to persons with Covid-19.

The laboratory investigations conducted in this article encompassed a range of essential tests, including complete blood count (CBC), CRP, ESR, lactate dehydrogenase (LDH), ferritin, and assessments of liver and renal function. Additionally, all patients underwent bedside echocardiography; abnormalities detected were promptly evaluated by a pediatric cardiologist within the hospital setting. To exclude coexisting infections with COVID-19, diagnostic evaluations for other infectious diseases were performed, such as leptospira IgM, scrub typhus card test, dengue IgM Enzyme-linked immunoassay (ELISA)/RT-PCR, and blood culture for salmonella.

Clinical presentations in this study were classified to symptoms and signs such as fever, conjunctivitis, rash, abdominal pain, vomiting, diarrhea, strawberry tongue, lymphadenopathy, pharyngitis, and neurological manifestations. Cardiac manifestations found by echocardiography included transient valve regurgitation, reduced left ventricular ejection fraction, coronary artery abnormalities, and pericardial effusion. All patients received treatment with intravenous immunoglobulin (IVIG) (2 g/kg) and aspirin (30–50 mg/kg). Patients with relapsed fever were treated with methylprednisolone (MDP) (2 mg/kg). Those with coronary dilation and aneurysm received antiplatelet therapy. No patients required vasopressor support. The outcome in our study represented as recovery, death, and any complications presented during the hospital stay.

### Study size, participants

The eligibility criteria for patient enrollment in this study are as follows: (1) Confirmation of MIS-C diagnosis according to WHO; (2) admission to the hospital between May 2020 and October 2021; (3) age range of adolescents and children between 0 and 19. Patients with concomitant infections, negative SARS-CoV-2 antibodies (unless exposed to other COVID-19 patients), and newborns were excluded from this study. In total, our research analysis includes 25 patients. The study adhered to the ethical standards outlined in the Declaration of Helsinki and was conducted on human subjects. Approval for the study was obtained from the Ethical Committee at Damascus University’s Faculty of Medicine (ID: MD130623-112), and informed consent was obtained from their legal guardians.

### Statistical analysis

The data analysis in this study was conducted using MS Excel. Continuous variables were represented as count, median, interquartile range (IQR), mean, and standard deviation. Categorical variables were represented as counts and percentages.

## Results

A total of 232 cases of COVID-19 were documented at Children’s Hospital in Syria from May 2020 to October 2021. Among these cases, we observed 25 occurrences of MIS-C, which accounts for 10.77% of the total. Remarkably, only 6 patients (24%) tested positive for SARS-CoV-2 through PCR testing, while 19 patients (76%) showed positive results for SARS-CoV-2 IgG and prior exposure to the virus within their families but RT-PCR and serological tests yielded negative results for these individuals. The median age of the patients was 5.5 years (IQR: 5–6), with 72% (*n* = 18) being male, and 28% (*n* = 7) female. All patients had no pre-existing health conditions.

**Fig. 1:**
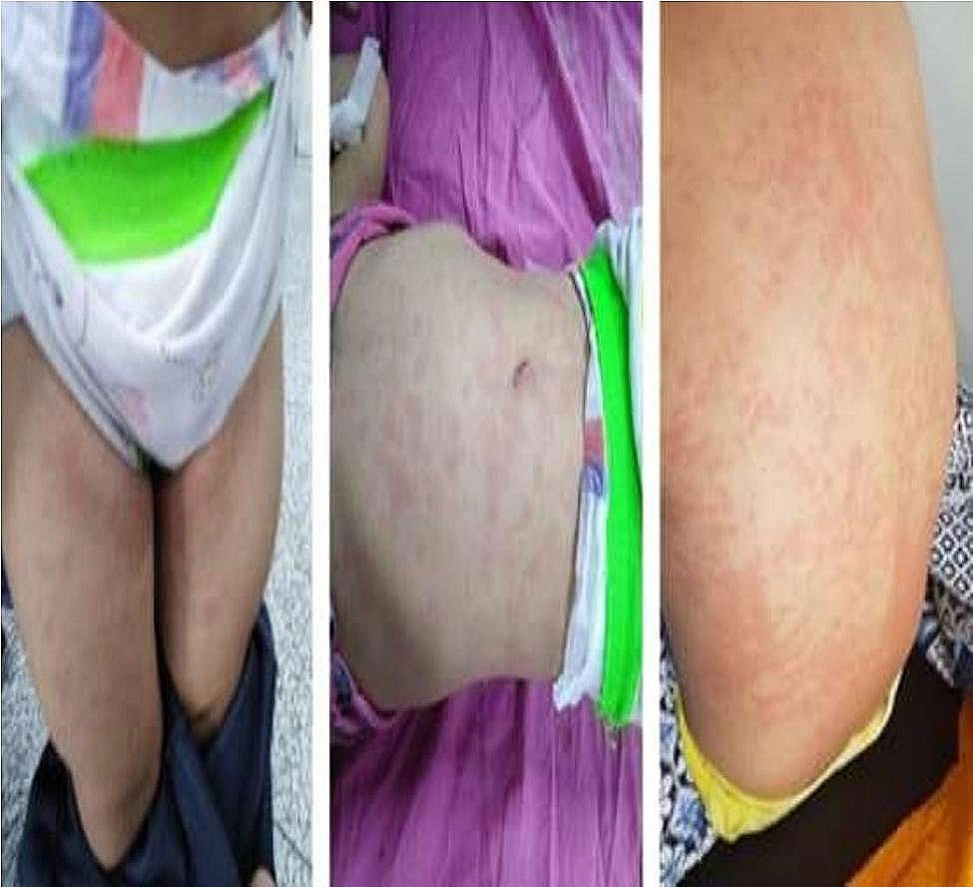
Patients with rash in trunk and extremities

**Fig. 2:**
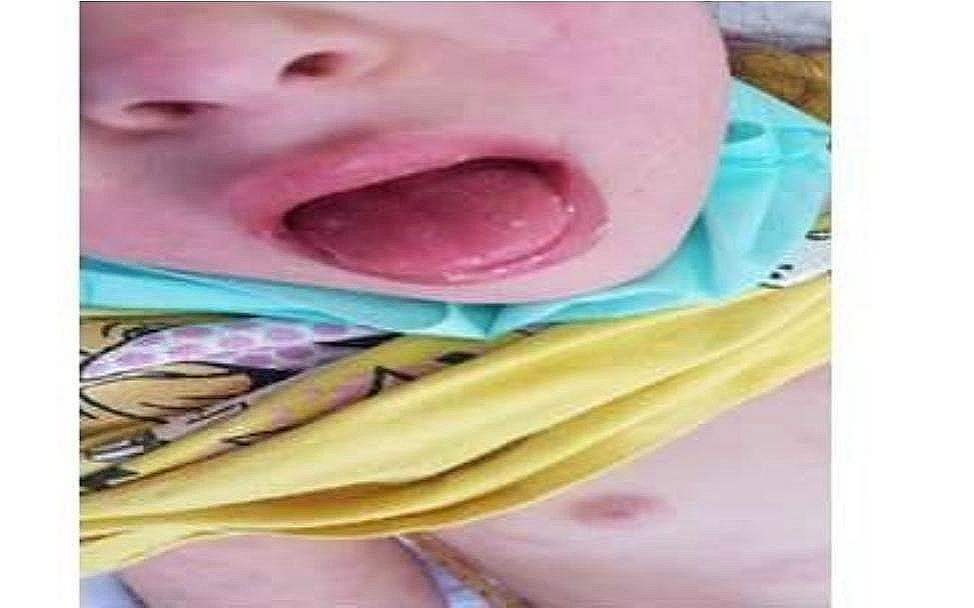
Patient with Strawberry Tongue

**Fig. 3:**
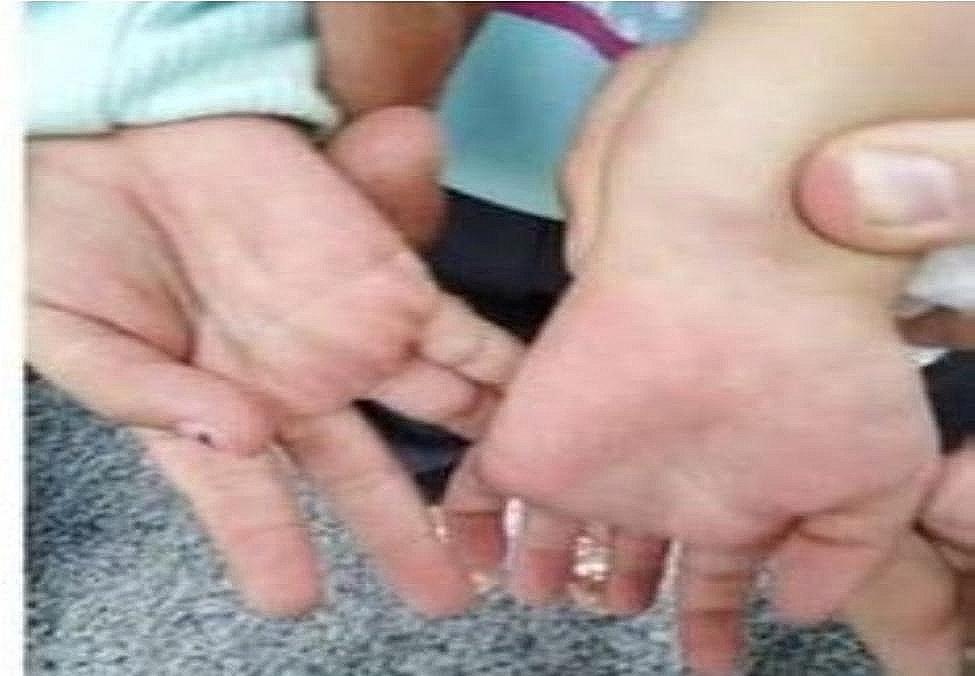
Patient with hands edema

All patients presented with fever (100%), bilateral conjunctivitis, and rash on the trunk and extremities (Fig. 1) (*n* = 22; 88% and *n* = 21; 84%), retrospectively. Gastrointestinal symptoms were observed in 19 patients (76%), including abdominal pain in 18 (72%), vomiting in 12 (48%), and diarrhea in 9 (36%). One patient was misdiagnosed with appendicitis and subsequently underwent appendectomy. Notable changes in the oral cavity were observed in 16 children (64%), such as strawberry tongue in 10 (40%) **(**Fig. 2**)** and pharyngitis in 9 (36%). Edema of the eyelids, hands, and feet was observed in 9 patients (36%) **(**Fig. 3**)**. Approximately nine patients (36%) exhibited cervical lymphadenopathy. Neurological manifestations were identified in 7 patients (28%), including one case of meningitis. Headaches were reported by a small number of patients (7; 28%). Respiratory symptoms were uncommon, occurring in only four patients (16%).

**Fig. 4:**
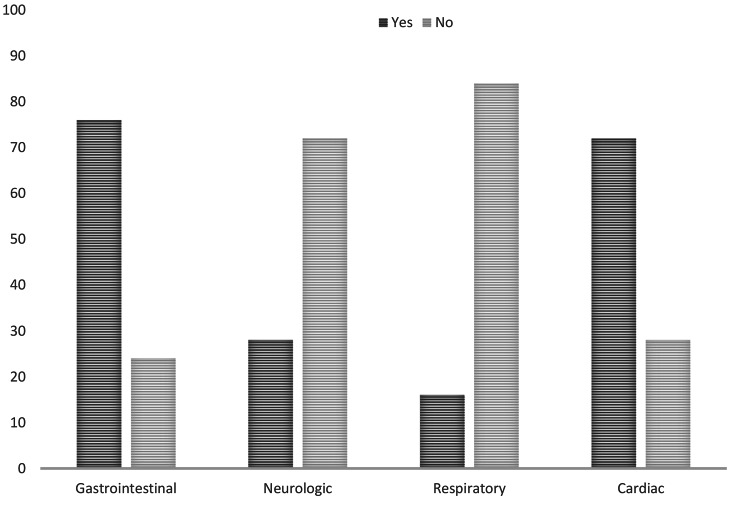
Bar chart illustrating the percentages of multi-systemic symptom occurrences

The majority of patients had cardiac dysfunction 18 (72%), including transient valve regurgitation 15 (60%), reduced left Ventricular ejection fraction 5 (20%), coronary artery abnormalities (dilatation and aneurysms), and pericardial effusion 4 (16%) each. Laboratory results are shown in Table 1. The percentages of multi-systemic symptom occurrences are illustrated in Fig. 4.

**Table 1:** Table showing values of the abnormal laboratory studies among patients

Laboratory Markers	N of patients (*n* = 25; %)	Mean (SD)	Normal Range	Laboratory Markers	N of patients (*n* = 25; %)	Mean (SD)	Normal Range
Leukocytosis (/cu.mm)	20 (80)	18,025 ± 2232	[4000-10,000]	Elevated ALT (U/L)	8 (32)	71.3 ± 11.5	[4–36]
Lymphopenia (/cu.mm)	24 (96)	1243 ± 130	[3000–9500]	Hypoalbuminemia (g/dl)	9 (36)	1.2 ± 1.4	[3.4–5.4]
Thrombocytopenia (/cu.mm)	10 (40)	99.4 ± 14.6	[150,000-400,000]	Elevated Ferritin (ng/ml)	25 (100)	873.8 ± 103.8	[7-140]
Elevated CRP (mg/l)	25 (100)	160.68 ± 25.4	[0.02–14.4]	Elevated Urea (mg/dl)	20 (80)	32.75 ± 15.09	[5-10]
Elevated ESR (mm/hr)	25 (100)	78.83 ± 14.3	[3-13]	Elevated Creatinine (mg/dl)	20 (80)	1.2 ± 0.19	[0.3–0.9]
Elevated D-dimer (ng/ml)	25 (100)	3,053.8 ± 1304.3	[90–560]	Elevated LDH (units/l)	15 (60)	534.2 ± 45.2	[143–370]

Abbreviations: CRP (C-reactive protein); ESR (Erythrocyte sedimentation rate); ALT (Alanine transaminase); LDH (Lactate dehydrogenase).

Regarding treatment, all patients received IVIG (2 g/kg) and aspirin (30–50 mg/kg), with approximately (86%) requiring continued treatment with methylprednisolone (MPD) (2 mg/kg) due to relapsing fever. Patients with coronary dilation and aneurysm (*n* = 4;16%) were given antiplatelet therapy.

All patients in this study recovered within 8 days of hospital stay, with no recorded death. One patient underwent appendectomy due to severe abdominal pain. A case of acute renal failure was reported in a patient after discharge from the quarantine department.

## Discussion

An epidemiological analysis conducted during the initial stages of the COVID-19 pandemic suggested that children were largely spared from the respiratory effects of the SARS-CoV-2 virus [11]. However, recent studies have revealed a higher prevalence of pediatric COVID-19 infections and increased risk of severe manifestations [12]. Pediatric COVID-19 infection is now recognized by the presence of multiple systemic symptoms and severe hyperinflammation [13] leading to the WHO defining this issue as MIS-C [10]. Among 232 COVID-19 cases admitted to our hospital, 25 individuals (10.77%) met the criteria for MIS-C.

Multiple syndromes have been characterized by severe inflammation and damage to multiple organs resulting from previous infections. While TSS is associated with bacterial superantigens produced by *Staphylococcus aureus* and *Streptococcus pyogenes* infections [14]MIS-C is triggered by the COVID-19 virus, which is supported by various epidemiological studies. These studies have shown a higher incidence of MIS-C in areas with a greater prevalence of COVID-19 infection [15]. It is worth noting that earlier studies indicated that 50% of MIS-C patients tested positive for SARS-CoV-2 through PCR testing [16–18]. In contrast, our study found a lower PCR-positive rate (24%) but a higher IgG-positive rate (76%), supporting MIS-C as a post-infectious condition [19]. The similarities in clinical and laboratory findings between MIS-C and other syndromes marked by hyperinflammation prompt inquiries into whether MIS-C is simply a variant of another syndrome or an independent clinical entity [15]. One syndrome that raises doubt on this hypothesis is KD. In fact, patients identified as having MIS-C were characterized as a variant of KD. However, there are significant differences in clinical features between KD and MIS-C. For instance, gastrointestinal symptoms occur in only 30% of KD patients, which is less than half the proportion seen in MIS-C patients in our study and other studies [15]. Additionally, laboratory findings also vary between these two syndromes. Although neutrophilia and leukocytosis are common in both conditions, KD is associated with an increase in platelet count, while in MIS-C, platelet count is slightly decreased or remains normal [15]. Our study also evidenced a low platelet count in 40% of patients and a normal count in the remaining individuals, supporting these findings.

MIS-C predominantly presents with fever, rash, and gastrointestinal problems. In our study, all patients had fever, 84% had a rash on their trunk and extremities, and 76% experienced gastrointestinal symptoms [15]. Among the gastrointestinal symptoms, abdominal pain was the most prevalent, affecting 78% of patients. Notably, one patient with this symptom was initially misdiagnosed with appendicitis and underwent an unnecessary appendectomy, underscoring the significance of accurately identifying COVID-19 patients and their atypical manifestations to prevent misdiagnosis. This was also highlighted in a previous article where abdominal pain was found to be severe, mimicking appendicitis and leading to unnecessary emergency surgeries [20]. Furthermore, our study revealed that MIS-C patients with cardiac involvement exhibited similar manifestations to those observed in patients with KD [15]. These manifestations included transient valve regurgitation, coronary artery abnormalities such as dilatation and aneurysms, and pericardial effusion, as described in our article [21]. However, coronary artery involvement in MIS-C cases was less common than in other cardiac conditions, with only four patients demonstrating this complication in our study [22]. Compared to adults, pediatric COVID-19 patients demonstrated fewer respiratory manifestations, as supported by previous studies [23]. Similarly, MIS-C patients also displayed fewer respiratory symptoms, with only 50% reporting such manifestations [24]. However, in our study, only 16% of patients presented with respiratory symptoms. This could be attributed to decreased expression of viral entry mediators in the respiratory epithelium and disparities in immune system responses between children and adults [25].

The management of MIS-C patients remains a topic of ongoing controversy. Multiple published guidelines emphasize the crucial importance of early identification and effective management of this syndrome [15]. The Royal College of Paediatrics and Child Health recommends providing supportive care to these patients, which includes close monitoring of hydration, vital signs, electrolytes, and metabolic status [15, 26]. It is generally recommended to administer aspirin, IVIG, and steroids to these patients as done in this study [15]. Furthermore, in line with the guidelines provided by the American Heart Association (AHA) for treating patients with KD [27], all patients in our study received aspirin treatment, and antiplatelet therapy was prophylactically prescribed for four patients with coronary artery aneurysm and dilation. The mortality rate associated with this syndrome is well-documented to be below 2%, with the majority of patients achieving full recovery [3]. Our study aligns with these findings, as no mortality was observed among the patients. These results highlight the favorable prognosis of this syndrome when managed according to recommended protocols.

There are certain limitations inherent in our study. Initially, data were collected from medical records in paper format, which may have led to the exclusion of certain data. Moreover, our research lacks a control group comprising COVID-19 patients who did not develop MIS-C, preventing the evaluation of potential risk factors in the onset of this syndrome. This underscores the necessity for further studies to address this gap in our article. The varying understanding among physicians regarding MIS-C may have resulted in the omission of relevant tests to evaluate its presence according to the defined criteria, potentially leading to missed cases. Lastly, the relatively small sample size of our study might impact the generalizability of our findings.

## Conclusion

The emergence of MIS-C as a distinct post-infectious syndrome associated with COVID-19 has been evident in epidemiological studies showcasing a higher incidence of MIS-C in areas with a greater prevalence of COVID-19 infection, This aligns with the hypothesis that MIS-C is triggered by the virus. The prevalence of positive SARS-CoV-2 IgG among our patients further substantiates the post-infectious nature of MIS-C. The clinical presentation of MIS-C includes fever, rash, and gastrointestinal symptoms, with abdominal pain being notably predominant. Cardiac involvement in MIS-C is similar to KD, but there are distinct differences in clinical features and laboratory findings. Respiratory symptoms are less common in both pediatric COVID-19 patients and MIS-C patients compared to adults. The management of MIS-C is subject of ongoing debate; however, early identification and supportive care are deemed pivotal. Yet, further research and larger studies are needed to establish more definitive guidelines for the management of MIS-C. Future research should address the limitations acknowledged in this study, offering a deeper understanding of MIS-C in pediatric COVID-19 patients.

## Data Availability

The datasets used and/or analysed during the current study available from the corresponding author on reasonable request.

## Abbreviations

COVID-19: Coronavirus disease 2019
MIS- C: Multisystem Inflammatory Syndrome in Children
KD: Kawasaki disease
TSS: toxic shock syndrome
MAS: macrophage activation syndrome
WHO: World Health Organization
PTT: Partial thromboplastin time
PT: Prothrombin time
CRP: C-reactive protein
ESR: Erythrocyte sedimentation rate
CBC: Complete blood count
LDH: lactate dehydrogenase
RT-PCR: Reverse transcription polymerase chain reaction
ELISA: Enzyme-linked immunoassay
IVIG: Intravenous immunoglobulin
MDP: methylprednisolone
